# Can Thermoclines Be a Cue to Prey Distribution for Marine Top Predators? A Case Study with Little Penguins

**DOI:** 10.1371/journal.pone.0031768

**Published:** 2012-04-20

**Authors:** Laure Pelletier, Akiko Kato, André Chiaradia, Yan Ropert-Coudert

**Affiliations:** 1 Université de Strasbourg, IPHC, Strasbourg, France; 2 CNRS, UMR7178, Strasbourg, France; 3 Research Department, Phillip Island Nature Parks, Cowes, Victoria, Australia; Institute of Marine Research, Norway

## Abstract

The use of top predators as bio-platforms is a modern approach to understanding how physical changes in the environment may influence their foraging success. This study examined if the presence of thermoclines could be a reliable signal of resource availability for a marine top predator, the little penguin (*Eudyptula minor)*. We studied weekly foraging activity of 43 breeding individual penguins equipped with accelerometers. These loggers also recorded water temperature, which we used to detect changes in thermal characteristics of their foraging zone over 5 weeks during the penguin’s guard phase. Data showed the thermocline was detected in the first 3 weeks of the study, which coincided with higher foraging efficiency. When a thermocline was not detected in the last two weeks, foraging efficiency decreased as well. We suggest that thermoclines can represent temporary markers of enhanced food availability for this top-predator to which they must optimally adjust their breeding cycle.

## Introduction

During reproduction, parents have to make decisions to optimise energy acquisition to simultaneously address their own needs and that of their offspring [1]. To this end, breeding animals should optimally match their peak of food requirements with the seasonal peak of resource availability [2]. A mismatching of these peaks can cause a decrease in the current reproductive output, as well as a reduction in the animal’s long-term fitness [3], [4]. The impact of this match-mismatch is particularly significant in marine ecosystems. The open ocean is a heterogeneous environment that is characterized by patchy prey distribution over a large time and spatial scale. As a consequence, top predators target places of high prey abundance, the hot spots which are a result of physical processes, such as up-wellings, eddies, gyres or sea-ice edges [5]. These places often change seasonally, annually or on a decadal basis [6]. Prey availability in these places can be affected by changes in oceanographic conditions, which could affect foraging success of marine top predators. These changes taking place at local foraging zones are generally influenced by large-scale processes [7] and can trigger a mismatch between predators and their prey.

For diving marine animals, oceanographic conditions of the water column, such as the presence of a thermocline, can also be important for the distribution of prey [8]. Clupeids, an abundant food source for top predators, can aggregate around thermoclines as shown, for example, in Argentine anchovies (*Engraulis anchoita*), which distribute preferentially in the layer immediately above the thermocline [9]. This is probably because thermoclines are rich in nutrients where the different levels of the food web concentrate [10], [11], [12]. For instance, the foraging behaviour of thick-billed murres (*Uria lomvia*) varies with the vertical distribution of prey, which is associated with annual variation in the intensity of the thermocline and water temperature at different depths [13]. Another seabird, Rhinoceros auklets (*Cerorhinca monocerata*) usually dive above or around the thermocline, indicating that either the distribution of their prey is constrained by this shift in temperature [14] or that the escape speed of the ectothermic prey is slowed down by the sudden change in temperature, making them easier targets to predators.

Here, we studied the foraging behaviour of the little penguin (*Eudyptula minor*), a marine diving seabird in which case the link between thermocline and foraging success has also been reported [15]. These authors found that a reduction in thermal stratification in the water detected by data loggers in a weak El Niño year (2006) was associated with reduced foraging success of little penguins. Thus, the increase in the mixing of the water column could have resulted from an increase in the wind force and in the number of storms [15], although other physical factors may lead to a similar mixing. The foraging patterns of penguins suggested that their prey were dispersed widely in the presence of poorly stratified waters [15]. In these studies [13]–[15], the absence of the thermocline reduced their foraging success during chick rearing, leading to a decrease in reproductive success. While previous studies [13]–[15] have looked at a composite of breeding/foraging success in relation to predominant oceanographic conditions over a whole season [16], no study, to our knowledge, has investigated the rate of prey encounter in relation to oceanographic conditions over short time scale (i.e. within a season).

In this study, we examined changes in the foraging activity and efficiency of breeding little penguins, while simultaneously monitoring changes in the vertical thermal characteristics of the water in their foraging zone. Since thermoclines can act as a boundary to prey distribution seasonally [15], we hypothesised that the presence of a thermocline could be a reliable signal of resource availability. We deployed miniature accelerometers on little penguins at early chick-rearing phase in a single season of high breeding success, when food supply was probably not a limiting factor [17]. We expect the ability of penguins to match the energetically demanding chick-rearing phase [18] with the presence of a thermocline to be critical to the foraging behaviour of these diving seabirds.

## Materials and Methods

The study was conducted on the little penguin breeding colony at Phillip Island (38°31′S, 145°09′E), Victoria, Australia. We deployed data loggers on 43 adult penguins at guard phase, tending chicks aged 1 to 2 weeks. At guard phase penguins make one-day foraging trips within 20 km from the colony [19]. The study period spanned 5 weeks, from 13 November to 17 December 2005. We used 12-bit, 52×15 mm, four-channel data loggers that weighed 16 g (M190L-D2GT, Little Leonardo, Tokyo, Japan) to record depth (resolution 0.05 m) and temperature (0.01°C) every second. This logger also recorded two axis accelerations along the longitudinal body axis (surging) and the dorso-ventral axis (heaving) of the bird, between −30 and 30 m s^−2^ at 32 Hz. The accelerometer measured both specific acceleration (e.g. movement) and gravity-related acceleration (e.g. posture).

Penguins were captured in their artificial nest box and loggers were attached on the lower back of the bird with Tesa tape [20]. All birds were recaptured in their nest boxes, the logger retrieved and the tape completely removed. Attachment and removal of the logger was completed within 5 min from the capture, and birds were returned to their nest-boxes. All equipped birds were monitored until the end of breeding [21]. Fieldwork protocol was approved by the Animal Experimentation Ethics Committee, Phillip Island Nature Park (PINP AEEC, number PINP AEEC 2.2004) with a research permit issued by the Department of Sustainability and Environment, Flora and Fauna (number 10003419) of Victoria, Australia.

Data were downloaded from the loggers into a computer and analysed using Igor Pro (Wavemetrics Inc., USA, 2008, Version 6.04). Given the low accuracy of the depth sensors at surface, only dives >1 m were considered for analysis [22]. Dive depth, total number of dives, time spent underwater, defined as the sum of all dive durations, and proportion of time at the bottom phase were calculated for each individual. A dive started and ended when birds departed and returned to the water surface. Start and end of bottom phases were defined as the first and last time the depth change rate became <0.25 m s^−1^ during a dive [22]. It is during the bottom phase of dives that little penguins encounter most of their prey (75.4%) [23].

We measured foraging efficiency using frequency and amplitude of flipper beatings, which were automatically extracted from the signal using purpose-written macro in Igor Pro (Wavemetrics Inc., USA, Version 4.02) [24]. The acceleration data were separated into low and high frequency components using the IFDL package from Igor [22]. Each propulsive stroke was recorded on the heaving axis resulting in a forward acceleration recorded on the surging axis. The amplitude of each stroke was analysed using the heaving acceleration, which is the most sensitive signal to detect frequency of strokes [22]. We could then identify periods of higher than the normal amplitude values observed during diving periods. Those periods of high amplitude were used as a proxy of prey encounter and pursuit [23]. Note this method does not provide a direct measure of prey consumption but prey encounter, which gives an estimate of food available to a bird during a trip. We calculated the ratio of the number of dives with prey encounter to the total number of dives during the foraging trip, as an index of hunting efficiency [24]. We determined prey encounter rates for depths >10 m because the high buoyancy of the birds in <10 m depth influences the flipper beating activity [15], [23].

Given that temperature sensors have a delayed time response (T_0.9_ ≈ 15 sec) [14], we corrected the temperature associated to depth following Daunt *et al.* [25]. Within a day trip, we determined a single thermal profile of the water column for each bird. For each hour of the day, we obtained a temperature profile by grouping temperatures from the same depth from both the descent and the ascent phase of the dive [15] during the course of the deepest dive (>25 m) [14]. From the several profiles obtained from a given bird, we calculated a mean temperature every 2 meters. This resulted in one thermal profile for each penguin that we then used to determine the presence or absence of a thermocline, defined as a zone of rapid decrease of temperature in the water column [26] in the five weeks of this study. The depth of the thermocline was visually detected from the vertical profile of temperature.

Statistical analysis of dive parameters was performed using the R software (version 2.8.1) [27]. For the hunting efficiency analyses, the sample size was only 39 birds due to missing data or excluding birds, which did not dive deeper than 10 m. We tested for normality and applied a logarithmic transformation when necessary. We used a generalized linear mixed model (GLMM) [28] with individuals as a random factor. For proportions, a binomial distribution was used, while a Poisson distribution was used for other variables. Subsequently, multiple comparisons were undertaken using the Tukey’s post hoc test. Unless otherwise stated, values are presented as mean ± SE with significance at 0.05.

## Results

**Figure 1:**
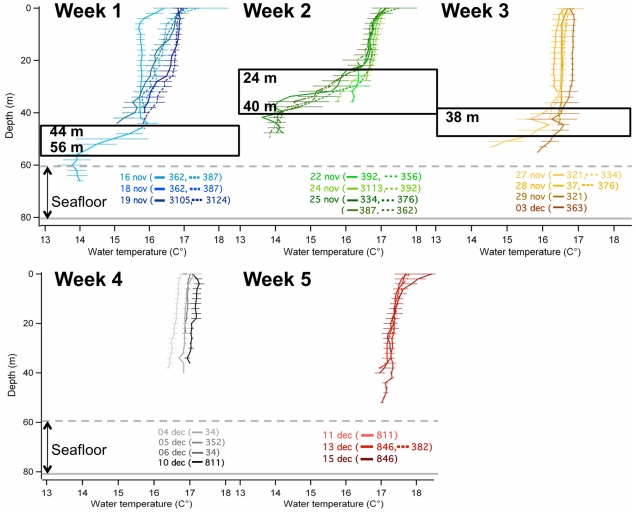
Changes of the thermal profiles of the water column in Bass Strait, Australia. During five weeks in November-December 2005, as measured by little penguins equipped with data loggers during one-day trips at their guard phase of breeding. Each temperature profile corresponds to a mean water temperature (±SD) every 2 meters calculated from several profiles obtained from each given bird (identified by date and nest number). The thermocline is framed in bold. The seabed is situated between 60 m (dotted, horizontal grey line) to 80 m (solid, horizontal grey line). We only represented the dive/temperature profiles of those birds that dived deeper than 25 m (see Materials and Methods for details).

All equipped birds made one-day trips and succeeding in raising their chicks until fledging. During the five weeks of the study, significant changes in the thermal profiles were observed in the water column (Figure 1). A thermocline was visible in the first three weeks, but not detected in the last two weeks. The thermocline was higher in the water column during the second week (24–40 m) compared with the first (44–56 m) and third weeks (38 m - the end of the thermocline being not detected). During the third week, the thermocline disappeared gradually from temperature profiles.

**Table 1:** Comparison of different diving parameters of little penguins during one-day trips at guard phase of breeding.

	Week 1	Week 2	Week 3	Week 4	Week 5
	(*n = 6*)	(*n = 12*)	(*n = 10*)	(*n = 7*)	(*n = 8*)
Nb of dive	892±127^a^	600±37^b^	979±140^c^	1165±140^a^	1441±149^a^
Dive depth (m)	10.9±0.1^a^	10.9±0.1^a^	8.2±0.1^a,b^	6.3±0.1^c^	6.1±0.1^b^
Time underwater (h)	6.6±0.4^a^	4.4±0.3^b^	4.8±0.6^c,d^	4.9±0.6^b,c^	5.8±0.3^d^
Bottom phase (%)	35.2±1.8^a^	28.3±1.4^b^	34.2±2.4^a^	35.7±2.2^a^	37±0.7^a^

Values expressed in mean ±SE over the five weeks. a, b, c, d: letters indicate significant differences (at 0.05). n = number of birds.

The mean number of dives performed by penguins during a foraging trip varied on a weekly basis (Table 1). Birds foraging during the second week made significantly less dives than individuals from other weeks (Tukey’s post hoc test: all p-values <0.05, Table 1). Moreover, deep dives (>25 m) were more frequent in the first two weeks (between 10 and 15% dives) than in the subsequent three weeks (4% of dives). A reduction in the mean dives depth was observed after the third week (Table 1).

**Figure 2:**
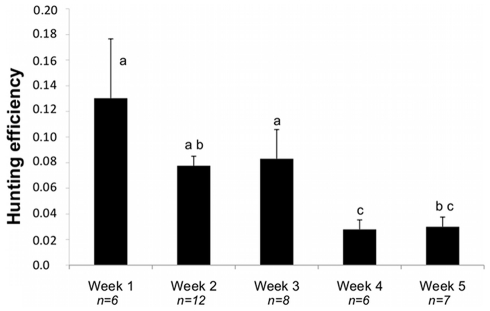
Changes of the hunting efficiency during the five weeks. Mean hunting efficiency ±SE (see Materials and Methods) of penguins for each week is represented. Letters a, b and c indicate significant differences (0.05) following GLMM-binomial and Tukey’s post hoc tests. n = number of birds.

The total time spent underwater also differed weekly (Table 1). During the first week, birds spent significantly more hours underwater than individuals in all other weeks (Tukey’s post hoc test: all p-values <0.05). For the second week, birds spent on average less time underwater, but also less time (in proportion) at the bottom phase of dives (Table 1). The time spent at the bottom phase of dives was equivalent for birds foraging the other four weeks (Table 1, Tukey’s post hoc test: all p-values >0.05). The hunting efficiency was higher for the first three weeks than for the last two (Figure 2), although the average efficiency of individuals from week 2 and week 5 was not significantly different (p-value = 0.056).

## Discussion

The thermal stratification of the water column in the foraging zone of little penguins changed over the course of the chick-rearing phase. These changes coincided with a decrease in foraging performance over time. Penguins showed higher hunting efficiency in the first 3 weeks when the thermocline was detected in the water column. Hunting efficiency declined while the total number of dives tended to increase when the thermocline weakened or was no longer detected.

No thermocline was detected in the temperature profiles recorded by the data loggers in the last two weeks of our study. A possible explanation for this is that the thermocline was deeper and not reached by penguins in the last two weeks. This is, however, unlikely given that little penguin can dive up to 70 m [29], implying that they are capable of foraging throughout the whole water column of Bass Strait with the mean depth between 60–80 m [30]. In week 5 for instance, birds dived as deep as 70 m, without detecting a thermocline. In fact, it would be surprising if the thermocline is located below 70 m, i.e. only 10 m above the maximum seabed depth in the penguin’s foraging zone. In any case, >70 m depth would be beyond the penguin’s reach. Secondly, birds could be foraging above the thermocline so that changes in water temperature were not detected by the data loggers. However, our biological data do not support that since penguins had lower hunting efficiency in weeks 4 and 5, suggesting that their foraging conditions were similar to those observed when thermoclines were absent. For these reasons, we suggest that the temperature profile recorded by the loggers were a close to real representation of the thermal structure in penguins foraging area over the course of our study (Figure 1).

When the thermocline was present in the water column, birds showed a higher hunting efficiency than when the thermocline was absent. Thermoclines are known to aggregate marine life. For example, anchovies, a common prey for little penguins [31], are known to concentrate around thermoclines [9]. While we believe prey can still be found sporadically distributed in the water column, the thermocline may act as a physical barrier, preventing prey from dispersing. The ectothermic nature of fish could be one possible explanation for this behaviour. The abrupt cooling when crossing the thermocline would reduce prey metabolism and consequently their maximum escape speeds, thus making them easy prey to predators [15], [32]. Alternatively, a high concentration of fish above the thermocline could be as consequence of phytoplankton being concentrated in the upper water mass [10], [11], [12].

Interestingly, birds foraging during week 2 had a high prey encounter with the smallest diving effort (few dives, little time spent underwater, short bottom time), which coincided with the period where the thermocline was the shallowest in the water column. This suggests that prey were probably concentrated at shallow waters on week 2 so penguins had less diving effort to capture them. In contrast, penguins increased the number of dives in the last two weeks reflecting an increase in birds’ foraging effort. Despite of greater number of dives, the prey encounter was lower than the first three weeks. This lower foraging efficiency coincided with absence of a thermocline in the foraging zone of the penguins towards the end of guard phase. In the absence of a thermocline, prey were likely to be more dispersed in a mixed water column so penguins were exploiting a less optimal environment.

Many seabird species can increase their foraging range and decline foraging success as the breeding season progresses. This change in foraging behaviour can be explained by prey depletion within the foraging zones close to the colony, the so-called Ashmole’s halo effect [33]–[36]. However, this energy-limitation hypothesis does not always find support in the literature [37]. Our results suggest an alternative explanation for a shift in foraging behaviour of diving birds during breeding. The lower prey encounter rate in the foraging area as the breeding season progresses could be explained by changes in oceanographic conditions that limit access to prey. For little penguins and perhaps for most diving marine animals, the presence and abundance of prey is not only associated with their distance from the central place and prey depletion but also with factors that affect prey distribution and availability in the water column, such as a thermocline and its change over time.

The absence of the thermocline late in the breeding season indeed led to an increase in diving effort while reducing hunting efficiency. We know that earlier breeding onset of little penguins has been related to an increase in sea surface temperature (SST) 3–6 months prior to breeding [38]. An increase in SST is precisely what can lead to the formation of a thermocline because stratification is initiated when the water surface warms up and separates from much colder deep water [39]. We propose here that if individuals are indeed adjusting the onset of breeding using SST information before the reproduction, then these individuals could be in a position to match their peak of food demand to peaks of food availability, as defined by the presence of thermoclines in their foraging zone. One main condition for this would be the ability of these individuals to relate those thermal regimes with prey availability and this can come through the accumulation of breeding attempts, i.e. experience [24], [40].

In this context, future work should examine how individual’s characteristics, such as age or experience, influence the ability of penguins to match their peak of food requirement to the presence of a thermocline in their foraging environment. These are important parameters to assess climatic scenarios, such as the predicted increase in El Niño events in the next decades [41]. El Niño events could lead to a greater mixing of the water column and disappearance of thermocline, affecting the foraging patterns of marine predators that depend, as shown in this study, on these thermal structures to forage more successfully.
